# Supplementary material for: Broad Bactericidal Activity of the Myoviridae Bacteriophage Lysins LysAm24, LysECD7, and LysSi3 against Gram-Negative ESKAPE Pathogens
Source: Viruses. 2019 Mar 21;11(3):284. doi: 10.3390/v11030284 (PMC6466606; doi:10.3390/v11030284)
Supplement: Supplementary file 1 [file viruses-11-00284-s001.pdf]

Supplementary Materials

Table S1. Sequences of all primers used.

#	Target gene	Direction	5'-PrimerSequence-3'
1	LysAm24	Forward	**5'-**AAGAAGGAGATATACATATGGACATTTTAAAATTTAACTCTC**-3'**
		Reverse	**5'-**TGGTGGTGGTGGTGCTCGAGTTTTAAAAATAAATCCTTTTCTGC**-3'**
2	LysECD7	Forward	**5'-**AAGAAGGAGATATACATATGTTTAAATTATCTCAAAGAAGCA**-3'**
		Reverse	**5'-**TGGTGGTGGTGGTGCTCGAGTTTTGGATCTGGGTATTTGCT**-3'**
3	LysSi3	Forward	**5'-**AAGAAGGAGATATACATATGCAACTCTCAAGAAAAGGT**-3'**
		Reverse	**5'-**TGGTGGTGGTGGTGCTCGAGCTTTGGGTATACACTGTCAAGATA**-3'**
